# Meaning Making in Cancer Survivors: A Focus Group Study

**DOI:** 10.1371/journal.pone.0076089

**Published:** 2013-09-26

**Authors:** Nadia van der Spek, Joel Vos, Cornelia F. van Uden-Kraan, William Breitbart, Rob A. E. M. Tollenaar, Pim Cuijpers, Irma M. Verdonck-de Leeuw

**Affiliations:** 1 VU University, Department of Clinical Psychology, Amsterdam, The Netherlands; 2 Department of Clinical Genetics, Leiden University Medical Center, Leiden, The Netherlands; 3 Department of Psychiatry and Behavioral Sciences, Memorial Sloan-Kettering Cancer Center, New York, New York, United States of America; 4 Department of Surgery, Leiden University Medical Center, Leiden, The Netherlands; 5 VU University Medical Center, Department of Otolaryngology - Head and Neck Surgery, Amsterdam, The Netherlands; National Taiwan University, College of Medicine, Taiwan

## Abstract

**Background:**

Confrontation with a life-threatening disease like cancer can evoke existential distress, which can trigger a search for meaning in people after having survived this disease.

**Methods:**

In an effort to gain more insight in the meaning making process, we conducted four focus groups with 23 cancer survivors on this topic. Participants responded to questions about experienced meaning making, perceived changes in meaning making after cancer and the perceived need for help in this area.

**Results:**

Most frequently mentioned meaning making themes were *relationships* and *experiences*. We found that, in general, cancer survivors experienced enhanced meaning after cancer through *relationships, experiences, resilience, goal-orientation* and *leaving a legacy*. Some participants, however, also said to have (also) experienced a loss of meaning in their lives through *experiences, social roles, relationships* and *uncertainties about the future*.

**Conclusions:**

The results indicated that there is a group of cancer survivors that has succeeded in meaning making efforts, and experienced sometimes even more meaning in life than before diagnosis, while there is also a considerable group of survivors that struggled with meaning making and has an unmet need for help with that. The results of this study contribute to develop a meaning centered intervention for cancer survivors.

## Introduction

Although cancer is still a leading cause of death worldwide, increasing numbers of people are being treated successfully [1]–[2]. About half of the cancer patients in developed countries recover from cancer and become long-term survivors [3]–[4]. Cancer survivors are at increased risk for serious psychological distress [5]–[6] and often struggle with unmet psychosocial needs [7]. Given that there will be more and more cancer survivors, a different approach in psycho-oncology will be needed to serve a novel target group: people who survive cancer and then deal with the sequelae that threaten their psychological well-being.

While some cancer survivors find it hard to cope with the psychosocial consequences of cancer and deal with existential issues like fear of death, isolation, rejection, meaninglessness, life questions and threats to self-identity, others experience hardly any problems in dealing with the aftermath of their disease. Some even report improved psychological well-being after cancer: they derive meaning from the cancer experience, feel more resilient, experience life more fully in the present or reprioritize their lives [8]–[13].

Confrontation with a life-threatening disease as cancer can evoke existential distress, which according to Lee [14], is the experience of life with little or no meaning. Lee [14] refers to the so called “existential plight of cancer” as the “search for meaning” following the cancer experience. Meaning in life is an important existential issue that is strongly related to psychological well-being and is liable to alteration after a negative experience like cancer [15]–[17]. The literature shows that some people clearly experience more meaning in life after they are confronted with cancer, whereas others adversely experience an absence or loss of meaning. Absence of meaning can lead to despair or demoralization and can trigger a search for meaning [18]–[21]. Several studies suggest that this search for meaning, or meaning making process, is only beneficial when meaning is found. Finding meaning is associated with psychological well-being, greater social adjustment, and less distress, while a continued search for meaning (without finding meaning) is negatively related to well-being, leads to higher levels of distress and is maladaptive [15], [22]–[26].

This is in line with the view of many existential psychotherapists, who consider meaning in life as a key concern in human existence, and assume that existential distress stems from failure in the search for meaning [27]. Frankl [28]–[30] states that the desire to find meaning is the primary and basic motivation of human beings. He developed *logotherapy*, an approach in psychotherapy that focuses on helping people to discover meaning or purpose in their lives and to overcome feelings of emptiness and despair.

Several studies have evaluated psychological interventions focusing on meaning making in cancer patients, mostly in the palliative phase. The majority of these studies show promising results with improved self esteem, optimism, mood, sense of meaning, spiritual well-being and decreased suffering after intervention [20], [31]–[36]. However, other studies did not report these improvements [37]–[38]. Research on this type of intervention is still in an early stage and much is unknown about meaning making and the possibilities of facilitating this process, especially in cancer *survivors*. A better understanding of meaning making after cancer can contribute to supporting cancer survivors more adequately in this process.

To obtain more insight in meaning making in cancer survivors, we conducted a focus group study. The study was designed to describe (1) the meaning making themes that play a role in cancer survivors, (2) the experienced changes in meaning making after cancer treatment and (3) the perceived needs for help in this particular area.

## Methods

### Study Design and Sample

A focus group method was chosen, because of its group dynamics which can provide rich data, especially when there is little prior knowledge [39]. Focus group participants were recruited in three different ways: (1) Research nurses recruited eligible patients at the outpatient clinic of an academic hospital; (2) psychologists in a mental health care institution that specializes in cancer patients approached eligible patients and (3) online advertisements were placed at websites from cancer patient organizations. Eligible patients were 18 years or older, diagnosed with cancer up to 7 years ago, treated with curative intent and were able to speak Dutch.

For this study, 37 persons showed interest to participate. Ten people were not available to attend the focus groups at the scheduled dates, or did not meet inclusion criteria. Each enlisted person was contacted by the researcher (NS) to make sure the participant met the criteria and to explain how and where the group would be conducted. Those who provided written informed consent, were scheduled to participate. Four people did not show up. Eventually, four focus groups were conducted in the summer and fall of the year 2011; three groups of six persons and one group of five participants (N = 23). The study has been approved by the Medical Ethical Committee of the Leiden University Medical Center, Leiden, the Netherlands. Characteristics of study participants are shown in *Table 1*.

**Table 1:** Characteristics of study participants (n = 23).

Characteristic	Number	%
*Sex*		
Male	7	30
Female	16	70
*Age*		
Mean (SD)	56 (11)	
Range	33–73	
*Type of cancer*		
Breast	10	43.5
Colorectal	8	34.8
Other (skin-, bone-, nose-, Hodgkin- and oesophagus cancer)	5	21.7
*Years since diagnosis* 1		
Mean (SD)	2 (1)	
Range	0.5–5	

1 There were no patients diagnosed longer than 5 years ago, who showed interest in participation.

### Procedure

Participants were sent information about the goals of the study and were asked to think beforehand about meaningful aspects in their lives and about whether there were physical or personal changes after cancer. The focus groups took place at VUmc University Medical Center in Amsterdam and LUMC University Medical Center in Leiden, The Netherlands, and each focus group lasted two hours. The groups were led by two moderators, a psychologist and a theologian, both researchers with experience in focus group moderation. The moderators followed a semi-structured moderating guide; topics are shown in *Table 2*. It was determined beforehand that a maximum of four focus groups could be conducted. After these four focus groups, data saturation was reached; meaning that no new information of value was obtained. All focus groups were audio taped and transcribed verbatim.

**Table 2:** Focus group topics and key questions.

Topics	Key questions
Meaning making	- What is meaningful in your life at the moment?
Changes in meaning making	- Did meaning in your life change after you were diagnosed with cancer? And if so, how did it change?
	- Have you ever had the feeling that you couldn’t find meaning? And how did you deal with that?
	- What helps you to find meaning, despite possible problems in your life?
Need for help with meaning making	- Are there aspects of meaning making that you wish you received help with? And if so, what kind of help would you like to receive?

### Analysis

Three coders independently analyzed the data following the steps of the “framework approach” [40]. First, they read all the transcripts thoroughly (*familiarization*). Key issues and underlying emerging themes were identified drawing on research questions that were posed a priori as well as issues that were raised by participant responses (*identifying a thematic framework*). The thematic framework was applied to all the data (*indexing*) and the data was rearranged according to the appropriate part of the thematic framework to which they relate (*charting*). The coders met regularly with a fourth researcher to resolve disagreements in coding. All data was analyzed by hand by the coders separately. Inter-rater reliability was substantial (*K* = .72) [41]. After that, consensus was reached on all disagreements, concepts were defined and data was interpreted (*mapping and interpretation*). We closely followed the Consolidated criteria for reporting qualitative research (COREQ) criteria; to gard the rigor of this qualitative study.

## Results

In this section we present the participants’ experiences with meaning making and closely related themes that emerged from the analysis of the transcripts. *Table 3* presents an overview of meaning making themes, perceived changes in meaning making (enhanced meaning and loss of meaning) and meaning-related issues after cancer mentioned in the focus groups.

**Table 3:** Meaning making themes and key-issues and themes of perceived changes in meaning making and meaning related issues.

**Sources of meaning**	Relationships (e.g. connection to friends and family)	
	Experiences (e.g. enjoying nature, going out to dinner with friends)	
	Creativity (e.g. painting, singing, writing)	
	Work (e.g. being successful, working in a team, contributing to society)	
	**KEY ISSUES**	**THEMES**
**Changes in meaning making**	**Enhanced meaning (through…)**	
	Relationships	- Meaning something to others
		- New or more intense relationships
	Experiences	- Experiencing life more intense
		- Enjoying (little things in) life more
		- Feeling more at ease
	Resilience	- Being more flexible towards uncertainties
		- Seeing things more in perspective
	Goal orientation	- Better able to prioritize
		- More balance in life
		- More decisive
	Leaving a legacy	
	**Loss of meaning (through)**	
	Experiences	- Loss of meaningful activities
		- Enjoying things less than before
		
	Social roles	- Unable to work anymore
		- Feeling incompetent as a parent or a partner
	Relationships	- Relationship problems with partner
		- Losing friendship
	Uncertainty about the future	- Hopelessness
	**Searching for meaning**	- Forced to shift to other sources of meaning
		- Still trying to find new meaning
	**Meaninglessness**	- Coping with meaninglessness through earlier experiences
**Meaning related issues**	- Isolation	
	- Threats to identity	
	- Physical limitations	
	- Confrontation with death	
	- Fear of passing cancer on to offspring	
	- Loss of freedom	

### Sources of Meaning

In the group discussions, it soon became clear that “meaning” is an abstract term that is not often used in daily Dutch language. Participants used other words for this experience, calling meaning *“a thrill”*, *“a sense of fulfillment”*, *“a kick”,* or *“the important things in life”*. Participants mentioned several meaning making themes (*Table 3*): meaning through relationships (e.g. feel more connected to family members), experiences (e.g. enjoy nature more), creativity (e.g. painting) and work (e.g. being successful).

### Changes in Meaning Making

#### Enhanced meaning

The majority of the participants mentioned to experience more meaning in life in certain specific areas. Many participants indicated to experience enhanced meaning through love and relationships with family and friends:

*“Meaning, you know….I just want **to be** there for people. How beautiful it is to be able to be there for someone. That has become my purpose.”*

Others described to experience enhanced meaning through experiences like enjoying the little things in life, like the sound of a bird or a good meal:

*“I enjoy the little things in life more and I live more in the present. I do not look as far ahead anymore, as it is of no use.”*

Most people noted to feel more resilient in dealing with adversities. Some people indicated that they became more goal-oriented live life more consciously and that they know better now what they find important in life.

*“I used to be a true workaholic, working 60, 70 hours a week. But I don’t do that anymore, it’s not worth it. Really, there are so many things I want to do. So many things I could spend my precious time on.”*

Some participants felt the need to leave a legacy. A person started writing a book to pass on his knowledge to his younger co-workers, some people started making photo albums, and someone else started a blog, as a sort of diary, for family and friends.

#### Loss of meaning

*Loss of meaning* was named less frequently by participants. Some participants mentioned a loss of meaning through experiences, because they werenot be able to continue their meaningful activities due to physical impairments, for example not being able to work, not being able to have children or not being able to do specific recreational, enjoyable activities anymore:

*“I have an invisible prosthesis in my leg, I can’t run anymore and lift heavy things anymore. I cannot make long walks. The treatment left me with neuropathy. That’s something you’re confronted with for every minute of your life.”*

*“I can’t do my job anymore. I am a physiotherapist, but I don’t have the strength to do my job anymore.”*

Some indicated that they do not enjoy some things less than they did before.

Other aspects participants named were not being able to fulfill certain important social roles in life (e.g. being a colleague or a good father) and loss of meaningful relationships.

*“Of course, something changes, because some people let you down, because they can’t or don’t want to talk about it [cancer]”.*

Some people experienced feelings of hopelessness because of uncertainties about the future. They experienced difficulties with setting goals and planning meaningful activities for the future.

#### Searching for meaning

In general, participants tried to keep sources of meaning the same as they were before diagnosis, but in some cases they felt forced to search for other sources of meaning:

*“I can do less things now, but the intensity has been shifted to other things. For example, things which used to give me satisfaction or purpose, it has been shifted from doings sports to…. To, like, enjoying the moment.”*

Others said they were still searching for a new meaning:

*“I used to get ideas and then I would just start. I can’t do that anymore. My artwork in the field, in the moment, that was where I got my thrill. And I don’t have that back yet. I can’t find it. I think I find it hard to accept that I can’t do as much physically.”*

#### Meaninglessness

When dealing with feelings of meaninglessness, many drew from experiences with meaning making in the past. Some had learned a lot about searching for meaning from another stressful event earlier in their lives, which made it easier to cope with cancer and derive meaning from it:

*“I had a burn out in 2008, which caused me to go into therapy for a year. That was more difficult than my cancer. That feeling returned for a while, but then you know: I can deal with this, but if you’ve never been in therapy, I can imagine that it hits you harder.”*

### Meaning Related Issues

Besides meaning making, several other existential issues were noted in the focus groups (*Table 3*). Of these other issues, isolation (loneliness) was most frequently named by the participants. Most mentioned that they had felt abandoned or misunderstood by others and that cancer has separated them in a way from the rest of their environment:

*“Yes, because after all the treatments, your hair starts growing and you carefully start working again. And the entire world goes: ‘hurray, she’s been cured’! And then it has to be finished.”*

*“People react so bluntly. ‘Yes, your breasts, you can just have those removed, right?’ Or ‘Oo, it didn’t get to your lymph nodes? O then it’s not too bad.’ Everyone has had enough of it. Your entire network has had enough of it, and you think: well I think it only just started now.”.*

Some participants indicated that they did not feel a connection with their social environment anymore. They often felt like an outsider among friends or co-workers.

*“Then you get back to work and people don’t really know how they should deal with you and I found that difficult, they almost ignore you because they don’t know what to say. It took me a year before I felt at ease again with others, before I could join in again. I’m still disappointed, but I do understand it.”*

Some struggled with threats to their identity. They felt that the new reality did not correspond to their self-image. This was mostly due to not being able to fulfill an old role (like parenting or working) in the same way anymore, or an experienced change in personal characteristics. One of the participants said:

*“I find it shocking… I used to be a person who remembered everything about everyone and now suddenly, not at all anymore. I forget things completely, it’s a total blank.”.*

Some felt that it is *other* people that view them differently:

*“It’s like you go to a party with people you don’t know. But they do know your partner, then you are ’ partner of…’. But you’re not, you’re just who you are. Before you know it, you are not ‘boss of the lab’ anymore, but ‘that man with cancer’”.*

Others found it hard to deal with their physical limitations; especially with the feeling that their body had let them down, and might do that again in the future:

*“At a certain point you hear the diagnosis: you have cancer. Well, what you hear is: I will die. It takes a while before you pass that. And I am. what I also found bothersome, you think you know your body. And you think your own body is tricking you. And it takes a while before you regain that trust.”*

Most participants indicated to be confronted with death at some point after diagnosis:

*“Recently I have experienced two funerals of two friends. So sometimes you realize that you’re lucky for still being around. We had cancer at the same time and… yes, death is quite confrontational. Then it really gets close to you.”*

A few people claimed they had barely thought of dying since they were diagnosed. Most people felt more awareness of the fact that life is ending and that you have no control over it.

*“I realized that I had always thought, without realizing,’ I will be, like, 80 years old’ that’s different now.”*

To some people, the thought of death evoked some anxiety, others felt relieved in a way that they had ‘gone through’ this fear of death:

*“Because yes, I have seen death, so… I don’t know how this was for you guys, but I have seen it. I absolutely don’t have fear of death anymore.”*

Some people indicated that they had a fear of passing the cancer on to their offspring.

One person mentioned that she experienced a loss of freedom, feeling like the cancer took control over her life and behavior.

*“Your self-confidence is completely shattered by something you can’t control. Cancer controls me, and I have no control over my life anymore.”*

### Perceived Need for Help with Meaning Making Issues in Cancer Survivors

The majority of the participants answered affirmative when asked if they needed help with meaning making. Most wanted help from a professional:

*“Someone who is unbiased to speak with”*

*“Someone who explains to you which emotional process you’re going through”.*

Others perceived a specific need for peer support, some specifically indicated that giving peer support to others is meaningful.

*“Fellow sufferers can help each other. You can be a companion for others. I think that that gives meaning.”*

A few people noted they had only felt a need for professional help, immediately after the diagnosis. They mentioned that it is important that the help is quickly accessible. Others disagreed and said that help was better suited about one and a half year after diagnosis and after treatment:

*“Once the storm is over, you start thinking: what happened to me in the past year? Not during the treatment”.*

Most participants agreed that when help with meaning making is offered, it should not be named as such. Many people felt offended by the link between meaning making and cancer, because they interpreted this as cancer being meaningful or that it should be considered as such. Some participants expressed a need for help for their partners, who according to most participants, do not get enough attention during the cancer process.

## Discussion

In this study, we investigated the perception of meaning making in cancer survivors. We found that, in general, cancer survivors experienced more meaning after cancer in at least one specific way, most frequently related to relationships and a newly found, more conscious way of living. Some participants, however, also mentioned to have (also) experienced a loss of meaning in their lives. These were mostly losses of meaning related to physical impairments or relational distress. In addition, it seemed that some people have an unmet need to fill a gap that arises from a loss of meaningful activities, for example not being able to work anymore.

The discrepancy in the literature between experiencing less or more meaning in life after cancer, was also shown in the outcomes of this study. Our results indicated that meaning making in cancer survivors is often a multifaceted process: in some specific areas (e.g. relationships) they experienced more meaning, while at the same time, meaning decreased in other areas (e.g. meaningful activities).

While this focus group study specifically aimed for more insight in meaning making processes in cancer survivors, also other related issues came up in the discussions. Many people stated that after they had been diagnosed with cancer they felt unacknowledged or abandoned in some way by most of their social environment, for example their co-workers, neighbors and other acquaintances. Some people seemed to miss a sense of belonging after having dealt with cancer, which can be seen as a characterization of the existential theme “isolation”, a term explained by Yalom [27] as a feeling of “separation from the world”. This finding corresponds with the theory of Ryff and Singer [42] that psychological well-being consists of two key dimensions: “leading a life of purpose” and “quality connections with others”.

A quality connection with others seemed to play a crucial role in the perception of the cancer survivors in this study. Close relationships with others were often mentioned as one of the most important sources of meaning, while the strongly related concept of isolation often came up as the hardest thing to deal with after cancer. An explanation of this seeming discrepancy could be that people derived meaning from the intimate relations they have with their beloved ones, like family members and close friends, but simultaneously feel more excluded from the rest of their social environment.

When asked if they had a need for help with meaning making since their diagnosis, most people confirmed that they had, confirming previous research indicating that cancer survivors have indeed unmet existential needs [43]. Participants also expressed a need for peer support. Some people considered supporting other peers to be meaningful. This finding relates to the “helper therapy principle”, a model by Riessman [43] that describes the therapeutic effect of giving and receiving support at the same time.

Also in line with previous studies [8]–[13], our results suggested that some people experience a satisfying, adaptive search for meaning, while others experience a continued, maladaptive search for meaning. Therefore, it is important to gain more knowledge on what the risk factors for meaning making problems are among cancer survivors, who may benefit from meaning making interventions and on how people with needs in this particular area can be screened and reached with interventions.

The results of this study did not only show that some people experience important shifts in meaning making, but also suggested that some meaning making needs are still unmet. Future psychological interventions should aim at these unmet needs.

### Strengths and Limitations

To our knowledge, there are no studies that used focus groups to investigate meaning making processes in cancer survivors. This study included a heterogeneous group of patients with various types of cancer to maximize the possibility of exploring a broad range of experiences and opinions from different perspectives. Although a valuable insight in patients’ experiences with meaning making was obtained, a few limitations should be noted.

The results are based on a relatively small sample size, which may hamper the generalizability. However, typically between four and six focus groups involving 4–10 participants is considered adequate [44]. Based on this study, no conclusions can be drawn on whether there were actual changes in meaning making after cancer, but only on whether these changes were *perceived*. Since meaning making is a personal, subjective process, we consider perceived changes more relevant than actual changes.

In addition, there were relatively many people in our sample that had already sought psychological help for coping with cancer. These people might struggle more in general than other cancer survivors and therefore also more with meaning making. This may give a biased view on experienced meaning making issues, however, it also sheds a light on a potential target group that might be at risk for meaning making problems. In this study, detailed information on participant characteristics, like marital status, education level, stage of cancer or type of treatment, was lacking. It is likely that these characteristics influence ones reflection on meaning making. This study was not set up to establish relations between variables, but mostly to generate ideas and explore different experiences with meaning making that cancer survivors might have.

In this study, we specifically asked about participant’s experiences and issues with meaning making. Without this specific asking, participants might not have mentioned these experiences, and this might have been influenced by the moderators, who were experts on this topic. The purpose of this study was not to objectively establish the themes that came to mind, but to gain more insight in the meaning making process specifically.

A critical point is that we did not obtain feedback of the participants on our findings to ensure the results are not curtailed by the researchers. However, the data was punctually transcribed verbatim, and coded and interpreted by three coders separately and discussed with a fourth researcher, to prevent curtailing.

The design of this study does not allow to draw conclusions about the prevalence of changes in meaning making in cancer survivors. Nevertheless, the results indicated that at least part of the cancer survivors clearly experience important shifts in meaning making after cancer.

*In conclusion* this qualitative study indicated that there is a group of cancer survivors that has succeeded in meaning making efforts, and experiences sometimes even more meaning than before diagnosis, while there is also a considerable group of survivors that struggles with meaning making and has an unmet need for help with that. These results may contribute to develop interventions targeting meaning in life in cancer survivors.

## References

[1] Brenner H (2002) Long-term survival rates of cancer patients achieved by the end of 20th century: a period analysis. Lancet, 1131–1135. 10.1016/S0140-6736(02)11199-8 12387961

[2] Richards MA, Stockton D, Bapp P, Coleman MP (2000) How many deaths have been avoided through improvements in cancer survival? Brit Med Journal, 895–898. 10.1136/bmj.320.7239.895 PMC27327 10741993

[3] Dutch Comprehensive Cancer Centres (2011) Cijfers over kanker. Kanker in Nederland. Availible: http://cijfersoverkanker.nl/. Accessed 12 March 2013.

[4] Howlader N, Noone AM, Krapcho M, Neyman N, Aminou R, et al. (2012) SEER Cancer Statistics Review, 1975–2009, National Cancer Institute. Available: http://seer.cancer.gov/csr/1975_2009_pops09/. Accessed 2013 Apr 9.

[5] Hoffman KE, McCarthy EP, Recklitis CJ, Ng AK (2009) Psychological Distress in Long-term Survivors of Adult-Onset Cancer Results From a National Survey. Arch Intern Med 14: 1274–1281. 10.1001/archinternmed.2009.179 19636028

[6] Carlson LE, Groff SL, Maciejewski O, Bultz BD (2010) Screening for distress in lung and breast cancer outpatients: a randomized controlled trial. J of Clin Oncol 28: 4884–4891. 20940193 10.1200/JCO.2009.27.3698

[7] Thewes B, Butow P, Girgis A, Pendlebury S (2004) Assessment of unmet needs among survivors of breast cancer. J Psychosoc Oncol 22: 51–73.

[8] Henoch I, Danielson E (2009) Existential concerns among patients with cancer and interventions to meet them: an integrative literature review. Psychooncology 18: 225–236. 18792088 10.1002/pon.1424

[9] Tedeschi RG, Calhoun LG (2004) Posttraumatic growth: Conceptual foundations and empirical evidence. Psychol Inq 15: 1–18.

[10] Thornton AA (2002) Perceiving benefits in the cancer experience. J Clin Psychol Med Settings 9: 153–165.

[11] Tomich PL, Helgeson VS, Nowak Vache EJ (2005) Perceived growth and decline following breast cancer: a comparison to age-matched controls 5-years later. Psychooncology 12: 1018–1029. 10.1002/pon.914 15744778

[12] Ferrell B, Smith SL, Cullinane CA, Melancon C (2003) Psychological Well Being and Quality of Life in Ovarian Cancer Survivors. Cancer 98: 1061–1071. 12942576 10.1002/cncr.11291

[13] Ussher J, Kirsten L, Butow P, Sandoval M (2005) What do cancer support groups provide which other supportive relationships do not? The experience of peer support groups for people with cancer. Soc Sci Med 62: 2565–2576. 16303220 10.1016/j.socscimed.2005.10.034

[14] Lee V (2008) The existential plight of cancer: meaning making as a concrete approach to the intangible search for meaning. Support Care Cancer 16: 779–785. 18197427 10.1007/s00520-007-0396-7

[15] Tomich PL, Helgeson VS (2002) Five years later: A cross-sectional comparison of breast cancer survivors with healthy women. Psychooncology 11: 154–169. 11921331 10.1002/pon.570

[16] Zika S, Chaimberlain K (1992) On the relation between meaning in life and well-being. Brit J of Psychol 83: 133–145. 1559144 10.1111/j.2044-8295.1992.tb02429.x

[17] Debats DL (1996) Meaning in life: Clinical relevance and predictive power. Brit J of Clin Psychol 35 503–516. 8955537 10.1111/j.2044-8260.1996.tb01207.x

[18] Lee CH (2006) Effects of Logotherapy with Exercise on meaning of life, ego integrity and IADL in the elderly. Taehan Kanho Hakhoe Chi 36: 701–709. 16953127 10.4040/jkan.2006.36.5.701

[19] Lee V, Cohen SR, Edgar L, Laizner AM, Gagnon AJ (2004) Clarifying “meaning” in the context of cancer research: a systematic literature review. Palliat Support Care 2: 291–303. 16594414 10.1017/s1478951504040386

[20] Lee V, Cohen SR, Edgar L, Laizner AM, Gagnon AJ (2006) Meaning-making intervention during breast or colorectal cancer treatment improves self-esteem, optimism, and self-efficacy. Soc Sci Med 62: 3133–3145. 16413644 10.1016/j.socscimed.2005.11.041

[21] Davis CG, Wortman CB, Lehman DR, Silver RC (2000) Searching for meaning in loss: Are clinical assumptions correct? Death studies 24 497–540. 11503666 10.1080/07481180050121471

[22] Park CL, Edmondson D, Fenster R, Blank TO (2008) Meaning Making and Psychological Adjustment Following Cancer: The Mediating Roles of Growth, Life Meaning, and Restored Just-World Beliefs.Cons and Clin Psy. 76: 863–875. 10.1037/a0013348 18837603

[23] Silver RL, Boon C, Stones MH (1983) Searching for meaning in misfortune: Making sense of incest. J of Soc Iss 39: 81–101.

[24] Thompson SC, Pitts J (1994) Factors relating to a person's ability to find meaning after a diagnosis of cancer. J of Psychosoc Onc 11: 1–21.

[25] Davis CG, Nolen-Hoeksema S, Larson J (1998) Making sense of loss and benefiting from the experience: two construals of meaning. J Pers Soc Psychol 75: 561–574. 9731325 10.1037//0022-3514.75.2.561

[26] Jaarsma TA, Pool G, Ranchor AV, Sanderman R (2007) The concept and measurement of meaning in life in Dutch cancer patients. Psychooncology 16: 241–248. 16850389 10.1002/pon.1056

[27] Yalom ID (1980) Existential psychotherapy (vol. 1 edn). Basic Books.

[28] Frankl V (1986) The doctor and the soul. From psychotherapy to logotherapy. London: Random House.

[29] Frankl V (1989) The will to meaning: foundations and applications of logotherapy (expanded edn). New York: Penguin Books.

[30] Frankl V (1998) Man's search for meaning: an introduction to logotherapy (British paperback edition) London: Random House.

[31] Chochinov HM, Hack TF, Hassard T, Kristjanson LJ, McClement S, et al (2005) Dignity Therapy: A Novel Psychotherapeutic Intervention for Patients Near the End of Life. J Clin Oncol 23: 5520–5525. 16110012 10.1200/JCO.2005.08.391

[32] Breitbart W, Rosenfeld B, Gibson C, Pessin H, Poppito S, et al (2010) Meaning-centered group psychotherapy for patients with advanced cancer: a pilot randomized controlled trial. Psychooncology 19: 21–28. 19274623 10.1002/pon.1556 PMC3648880

[33] Giese-Davis J, Koopman C, Butler LD, Classen C, Cordova M, et al (2002) Change in emotion-regulation strategy for women with metastatic breast cancer following supportive-expressive group therapy. Cons and Clin Psy 70: 916–925. 10.1037//0022-006x.70.4.916 12182275

[34] Bordeleau L, Szalai JP, Ennis M, Leszcz M, Speca M, et al (2003) Quality of life in a randomized trial of group psychosocial support in metastatic breast cancer: overall effects of the intervention and an exploration of missing data. J of Clin Oncol 21: 1944–1951. 12743147 10.1200/JCO.2003.04.080

[35] Classen C, Butler LD, Koopman C, Miller E, DiMiceli S, et al (2001) Supportive-expressive group therapy and distress in patients with metastatic breast cancer. Arch of Gen Psychiatry 28: 494–501. 10.1001/archpsyc.58.5.494 11343530

[36] Spiegel D, Spira J (1991) Supportive-Expressive Group Therapy: A treatment manual of psychosocial intervention for women with recurrent breast cancer. Stanford, CA: Stanford University School of Medicine.

[37] Kissane DW, Bloch S, Smith GC, Miach P, Clarke DM, et al (2003) Cognitive-Existential Group Psychotherapy for Women with Primary Breast Cancer: A Randomised Controlled Trial. Psychooncology 12: 532–546. 12923794 10.1002/pon.683

[38] Coward DD (2003) Facilitation of self-transcendence in a breast cancer support group: II. Onc Nurs Forum 30: 291–300. 10.1188/03.ONF.291-300 12692663

[39] Rabiee F (2004) Focus-group interview and data analysis. Procof the NutSoc 63: 655. 10.1079/pns2004399 15831139

[40] Pope C, Ziebland S, Mays N (2000) Analyzing qualitative data. BMJ, 114–116. 10.1136/bmj.320.7227.114 PMC1117368 10625273

[41] Landis JR, Koch GG (1977) An application of hierarchical kappa-type statistics in the assessment of majority agreement among multiple observers. Biometrics, 363–374. 884196

[42] Ryff CD, Singer BH (1998) Contours of positive human health. Psychol Inq, 1–18.

[43] Hodgkinson K, Butow P, Hunt GE, Pendlebury S, Hobbs KM, Wain G (2007) Breast cancer survivors’ supportive care needs 2–10 years after diagnosis. Support Care Cancer 15: 515–523. 17120068 10.1007/s00520-006-0170-2

[44] Litosseliti L (2007) Using focus groups in reserach. London: Continuum.

